# Cu- and Mn-bearing tourmalines from Brazil and Mozambique: crystal structures, chemistry and correlations

**DOI:** 10.1007/s00710-012-0234-6

**Published:** 2012-11-08

**Authors:** Andreas Ertl, Gerald Giester, Ulrich Schüssler, Helene Brätz, Martin Okrusch, Ekkehart Tillmanns, Hermann Bank

**Affiliations:** 1 Institut für Mineralogie und Kristallographie, Geozentrum, Universität Wien, Althanstr. 14, 1090 Wien, Austria; 2 Lehrstuhl für Geodynamik und Geomaterialforschung, Institut für Geographie und Geologie, Universität Würzburg, Am Hubland, D-97074 Würzburg, Germany; 3 Geozentrum Nordbayern, Universität Erlangen, Schlossgarten 5a, 91054 Erlangen, Germany; 4 Gebrüder Bank, Dietzenstrasse 1, 55743 Idar-Oberstein, Germany

## Abstract

Cu- and Mn-bearing tourmalines from Brazil and Mozambique were characterised chemically (EMPA and LA-ICP-MS) and by X-ray single-crystal structure refinement. All these samples are rich in Al, Li and F (fluor-elbaite) and contain significant amounts of CuO (up to ~1.8 wt%) and MnO (up to ~3.5 wt%). Structurally investigated samples show a pronounced positive correlation between the <*Y*-O> distances and the (Li + Mn^2+^ + Cu + Fe^2+^) content (apfu) at this site with *R* ^2^ = 0.90. An excellent negative correlation exists between the <*Y*-O> distances and the Al_2_O_3_ content (*R* ^2^ = 0.94). The samples at each locality generally show a strong negative correlation between the *X*-site vacancies and the (MnO + FeO) content. The Mn content in these tourmalines depends on the availability of Mn, on the formation temperature, as well as on stereochemical constraints. Because of a very weak correlation between MnO and CuO we believe that the Cu content in tourmaline is essentially dependent on the availability of Cu and on stereochemical constraints.

## Introduction

In tourmaline, which has the generalised structural formula *XY*_3_ *Z*_6_(BO_3_)_3_ *T*_6_O_18_ *V*_3_ *W*, the individual structural sites can be occupied by the following cations: site *X* = Na, Ca, K, □ (vacancy); *Y* = Li, Mg, Fe^2+^, Mn^2+^, Al, Cr^3+^, V^3+^, Fe^3+^, Ti^4+^, Zn, Cu; *Z* = Al, Mg, (Fe^2+^), Fe^3+^, Mn^3+^, V^3+^, Cr^3+^, (Ti^4+^); *T* = predominantly occupied by Si, but sometimes also by Al and B; *V* = OH^-^, O^2-^; *W* = OH^-^, F^-^, Cl^-^, O^2-^ (*e.g.,* Povondra and Čech 1976; Foit and Rosenberg 1979; Deer et al. 1986; Foit 1989; Grice and Ercit 1993; Hawthorne et al. 1993; Lussier et al. 2009, 2011; MacDonald and Hawthorne 1995a, b ; Hawthorne 1996; Henry and Dutrow 1996; Ertl et al. 1997, 2003, 2005, 2007, 2008, 2009, 2010a, 2012; Dyar et al. 1998; Hawthorne and Henry 1999; Hughes et al. 2001, 2004; Marler and Ertl 2002; Henry et al. 2011; Ertl and Tillmanns 2012). Substitutions at the *Y* site are more constrained by size than by valence; consequently the variety of cations is larger than on the *Z* site, where substitutions are more constrained by valence than by size (Grice and Ercit 1993).

The crystal structure of Cu-bearing tourmalines from Paraiba, Brazil, was described for the first time by MacDonald and Hawthorne (1995b). They refined two Cu-bearing elbaites, which contain 0.38 and 0.81 wt% CuO and 0.69 and 0.30 wt% Mn_2_O_3_, respectively. Another Cu-bearing elbaite (with 0.94 wt% CuO and 0.40 wt% Mn_2_O_3_) from the same locality was refined by Ertl et al. (2002).

The crystal structure of a Mn-rich tourmaline from Zambia, which contains a relatively high amount of Mn^2+^ (0.93 apfu), was described for the first time by Nuber and Schmetzer (1984). Burns et al. (1994) refined the crystal structures of eight Mn-bearing tourmaline samples (from Nepal, Zambia and the San Diego Mine, California, USA). These samples contained up to 6.23 wt% MnO. The crystal structure of Mn^2+^-rich tourmalines (with up to 8.66 wt% MnO) from Eibenstein an der Thaya, Lower Austria, was described by Ertl et al. (2003). Bosi et al. (2005a, 2012) published refinements of the crystal structures of Mn^2+^-rich tourmalines (with up to 9.6 wt% MnO) from the island of Elba, Italy.

Here we describe the crystal structure and the chemistry of tourmalines from Brazil and Mozambique, which contain up to 1.78 wt% CuO and up to 3.51 wt% MnO.

## Experimental

### Sample selection

#### Tourmalines from Brazil

Blue and green tourmalines from granite pegmatites in the vicinity of the village São José de Batalha near Salgandinho, Brazilian state of Paraiba, became available on the gem market in 1987, exciting considerable interest to gemmologists and gem traders. It turned out that the spectacular colours of these elbaites are due to the combined effect of Mn and Cu; the trace element Cu was hitherto not recorded in tourmaline (Bank et al. 1990; Henn et al. 1990; Fritsch et al. 1990; Henn and Bank 1990; Rossman et al. 1991). Interestingly, Brandstätter and Niedermayr (1993) detected inclusions of dendritical native copper in relatively Fe-rich Cu-Mn elbaites from São José de Batalha.

In their polarized absorption spectra, the blue Paraiba elbaites reveal strong, dichroic absorption bands with maxima at about 920 and 700 nm, caused by Cu^2+^, and at 520 nm, caused by Mn^3+^, both in distorted octahedral coordination, whereas the blue Fe^2+^-containing elbaites show the dichroic absorption band of Fe^2+^ at 710 nm (Mattson and Rossman 1988; Henn and Bank 1990; Shigley et al. 2001). Fe^2+^ also has a 2d absorption band that peaks near 1120 nm whereas Cu^2+^ does not have a band that peaks in this region (Rossman et al 1991). According to Rossman et al. (1991), the vivid yellowish green to blue green colours are due primarily to Cu^2+^ and are modified to blue and violet hues by increasing absorption from Mn^3+^. It is less likely that the presence of Fe^2+^ at a very low concentration could be responsible for a different colour in the samples because the colours from Fe^2+^ and Cu are nearly identical. Particularly, if the amount of Fe^2+^ is very low, the transmission window defined by both elements is very similar, and thus a small amount of Fe^2+^ will not have much effect on the color (Rossman, pers. comm.).

In an amethyst-coloured Paraiba tourmaline, Schultz-Güttler (2003) recognized an unusual inverse colour change from violet in daylight to blue in incandescent light, which he ascribes to specialities in the absorption intensities of Mn^3+^ and Cu^2+^.

Similar Cu-Mn-bearing, Paraiba type elbaites were recorded in granitic pegmatites at the nearby localities of Quintos de Baixo and Boquerão, in the state of Rio Grande del Norte (Karfunkel and Wegner 1996; Shigley et al. 2001; Milisenda 2005; Milisenda et al. 2006).

**Fig. 1:**
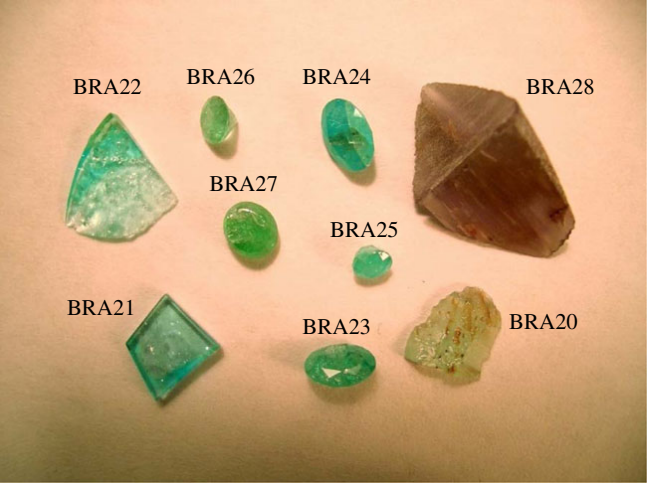
Investigated tourmaline crystals (raw and cut) from Brazil (BRA20-BRA28)

Under the designation “Brazil”, we investigated 9 samples chemically (Fig. 1) and 4 samples were characterised by single-crystal structure refinement. These samples display mostly blue, bluish green or yellowish green colours, typical of Paraiba elbaites. None of the Brazilian tourmalines investigated revealed optical zonation.

#### Tourmalines from Mozambique

Gem-quality tourmalines from the Alto Ligonha plateau, northern Mozambique, are known at least since 1953 (Henn and Bank 1997). They occur in Nb-Ta-Bi pegmatites of Pan-African age (about 500 Ma), which bear gem-quality minerals like beryl, spodumene and garnet (Hutchinson and Claus 1956; Henn and Bank 1991, 1997). A new occurrence of Cu-Mn bearing elbaites in the Alto Ligonha pegmatite province was detected in the Yuluchi Mountains, some 150 km SW of the city of Nampula. The stones are mined from placer deposits, but are presumably derived from pegmatites (Milisenda et al. 2006).

The Mozambique tourmalines display a wide variation in colours, i.e. violet, pink, purple, blue, greenish blue, yellowish green and green. Microprobe analyses on blue, bluish green and green crystals revealed Mn and Cu contents.

**Fig. 2:**
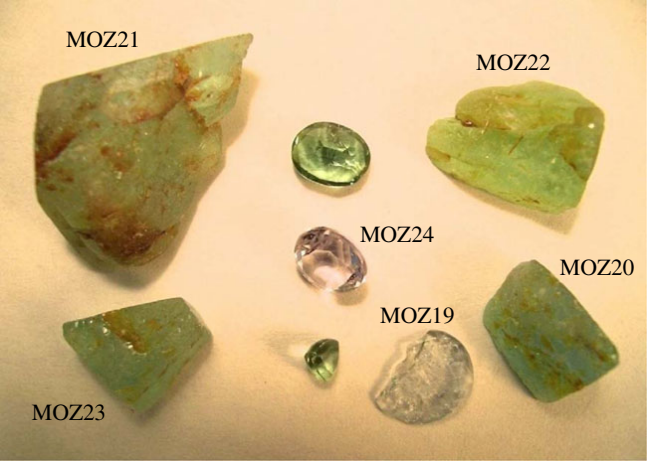
Investigated tourmaline crystals (raw and cut) from Mozambique (MOZ19-MOZ24)

We investigated 6 samples from Mozambique chemically (Figs. 2) and 4 samples were characterised by single-crystal structure refinement. These samples display a large variety of colours, i.e. violet-pink, blue, bluish green, pale green, yellowish green, greenish yellow. None of the tourmaline crystals investigated shows optical zonation.

### Chemical composition

#### Electron microprobe analyses

The tourmalines were analyzed for major and minor elements at Würzburg University. For EMPA a CAMECA SX 50 microprobe with three wavelength-dispersive channels was used. Analytical conditions were 15 kV accelerating voltage, 15 nA beam current, 10 μm beam diameter with regard to the measurement of F, and counting times of 20 seconds for most of the major elements, 30 seconds for Fe and Mn. Well-characterised natural and synthetic silicate and oxide mineral standards or pure element standards supplied by CAMECA were used. Kα radiation was taken for the analysis of F, Na, Al, Si, Cl, K, Ca, Ti, V, Mn, Fe, and Lα for the analysis of Ba. Special care was taken to account for overlapping peaks, especially V (Kα) with Ti (Kβ). The matrix correction of the EMPA data was done by the PAP program of CAMECA (Pouchou and Pichoir 1985). Using these analytical conditions, the detection limit is at 0.05 wt%. The analytical precision is <1 % relative for all major elements, <5 % relative for all minor elements and ≤10% relative for F.

As tourmalines may be very heterogeneous from a compositional point of view, we tried to get a larger part of the polished plane of the crystal into measuring position. Five single EMP analyses were carried out at distant points on the plane to recognize possible zonation which, however, was not detected. An apparent zonation in some tourmalines in Figs. 1 and 2 (e.g. BRA22), is caused by varying thickness of the samples. In Table 1, the average of the five analyses is presented.

**Table 1:** Composition of Cu- and Mn-bearing fluor-elbaite from Brazil (BRA) ad Mozambique (MOZ) (wt%)

	BRA20	BRA21	BRA22	BRA23	BRA24	BRA25	BRA26	BRA27	BRA28	MOZ19	MOZ20	MOZ21	MOZ22	MOZ23	MOZ24
SiO_2_	37.28	37.03	37.52	36.36	37.19	37.13	36.26	36.17	37.05	37.43	36.51	36.79	36.50	36.60	37.18
TiO_2_	<0.05	<0.05	0.05	<0.05	<0.05	<0.05	<0.05	0.07	<0.05	<0.05	<0.05	<0.05	0.05	<0.05	<0.05
B_2_O_3_	10.70^**^	10.80^**^	10.79^**^	10.70^**^	10.71^*^	10.70^*^	10.64^**^	10.54^**^	10.79^**^	10.72^**^	10.52^**^	10.70^*^	10.61^**^	10.72^**^	10.84^**^
Al_2_O_3_	40.69	42.44	41.60	41.26	40.73	40.37	40.21	38.83	42.36	41.26	39.39	41.19	40.72	40.96	43.13
MgO^*^	<0.01	<0.01	<0.01	<0.01	<0.01	<0.01	0.07	0.20	<0.01	<0.01	<0.01	<0.01	<0.01	<0.01	<0.01
CaO	0.78	0.10	0.20	0.31	0.75	0.80	0.34	0.26	0.11	0.71	0.56	0.10	0.21	0.10	0.08
MnO	1.45	0.54	0.62	2.01	0.40	0.97	2.54	3.19	1.01	0.49	3.09	2.47	2.34	3.51	<0.05
FeO	<0.05	<0.05	0.05	<0.05	<0.05	<0.05	0.21	<0.05	<0.05	<0.05	<0.05	<0.05	0.06	<0.05	<0.05
CuO^*^	0.33	0.81	0.88	1.27	1.78	1.69	0.95	1.57	0.26	0.30	0.35	0.19	0.15	0.17	0.12
ZnO^*^	<0.01	<0.01	<0.01	0.01	0.02	<0.01	0.72	0.63	<0.01	0.01	<0.01	<0.01	<0.01	<0.01	<0.01
PbO^*^	0.01	<0.01	<0.01	<0.01	0.01	0.01	0.01	<0.01	<0.01	0.14	0.01	<0.01	<0.01	<0.01	<0.01
BiO^*^	0.15	<0.01	<0.01	0.01	0.09	0.04	0.31	0.01	0.02	0.04	0.09	<0.01	0.01	<0.01	0.02
Li_2_O^**^	1.48	1.28	1.39	1.17	1.44	1.42	1.12	1.07	1.28	1.56	1.29	1.20	1.23	1.14	1.35
Na_2_O	2.06	2.10	2.09	2.25	2.15	2.24	2.38	2.56	2.07	1.99	2.25	2.24	2.32	2.40	2.04
K_2_O	<0.05	<0.05	<0.05	<0.05	<0.05	<0.05	<0.05	0.05	<0.05	<0.05	<0.05	<0.05	<0.05	<0.05	<0.05
F	1.33	1.07	1.29	1.22	1.77	1.69	1.58	1.53	1.18	1.39	1.69	1.27	1.36	1.19	0.91
H_2_O^**^	3.06	3.22	3.11	3.11	2.86	2.89	2.92	2.91	3.17	3.04	2.83	3.09	3.02	3.14	3.31
O≡F	-0.56	-0.45	-0.54	-0.51	-0.75	-0.71	-0.67	-0.64	-0.50	-0.59	-0.71	-0.54	-0.57	-0.50	-0.38
Sum	98.76	98.94	99.05	99.17	99.15	99.24	99.59	98.95	98.80	98.49	98.87	98.70	98.01	99.43	98.60
*N*	31	31	31	31	31	31	31	31	31	31	31	31	31	31	31
Si	6.06	5.96	6.04	5.91	6.04	6.04	5.93	5.97	5.97	6.07	6.03	5.97	5.98	5.93	5.96
^[4]^Al	0.00	0.04	0.00	0.09	0.00	0.00	0.07	0.03	0.03	0.00	0.00	0.03	0.02	0.07	0.04
Sum *T* site	6.06	6.00	6.04	6.00	6.04	6.04	6.00	6.00	6.00	6.07	6.03	6.00	6.00	6.00	6.00
^[3]^B	3.00	3.00	3.00	3.00	3.00	3.00	3.00	3.00	3.00	3.00	3.00	3.00	3.00	3.00	3.00
Al	7.79	8.00	7.90	7.81	7.79	7.74	7.67	7.52	8.00	7.88	7.67	7.86	7.84	7.76	8.12
Mg	-	-	-	-	-	-	0.02	0.05	-	-	-	-	-	-	-
Mn^2+^	0.20	0.07	0.08	0.28	0.06	0.13	0.35	0.45	0.14	0.07	0.43	0.34	0.32	0.48	-
Fe^2+^	-	-	0.01	-	-	-	0.03	-	-	-	-	-	0.01	-	-
Cu	0.04	0.10	0.11	0.15	0.21	0.20	0.11	0.19	0.03	0.04	0.04	0.02	0.02	0.02	0.01
Zn	-	-	-	-	-	-	0.09	0.08	-	-	-	-	-	-	-
Li	0.97	0.83	0.90	0.76	0.94	0.93	0.73	0.71	0.83	1.01	0.86	0.78	0.81	0.74	0.87
Sum *Y*, *Z* sites	9.00	9.00	9.00	9.00	9.00	9.00	9.00	9.00	9.00	9.00	9.00	9.00	9.00	9.00	9.00
Ca	0.14	0.02	0.04	0.05	0.13	0.14	0.06	0.05	0.02	0.12	0.10	0.02	0.04	0.02	0.01
Pb	-	-	-	-	-	-	-	-	-	0.01	-	-	-	-	-
Bi	0.01	-	-	-	-	-	0.01	-	-	-	-	-	-	-	-
Na	0.65	0.66	0.65	0.71	0.68	0.71	0.75	0.82	0.65	0.63	0.72	0.71	0.74	0.75	0.63
K	-	-	-	-	-	-	-	0.01	-	-	-	-	-	-	-
Vacancy	0.20	0.32	0.31	0.24	0.19	0.15	0.18	0.12	0.33	0.24	0.18	0.27	0.22	0.23	0.36
Sum *X* site	0.80	0.68	0.69	0.76	0.81	0.85	0.82	0.88	0.67	0.76	0.82	0.73	0.78	0.77	0.64
OH	3.32	3.46	3.34	3.37	3.09	3.13	3.18	3.20	3.40	3.29	3.12	3.35	3.30	3.39	3.54
F	0.68	0.54	0.66	0.63	0.91	0.87	0.82	0.80	0.60	0.71	0.88	0.65	0.70	0.61	0.46
Sum OH + F	4.00	4.00	4.00	4.00	4.00	4.00	4.00	4.00	4.00	4.00	4.00	4.00	4.00	4.00	4.00

All data by EMP analyses, except ^*^ by LA-ICP-MS on the tourmaline bulk samples. Average of 10 EMP analyses or 3 LA-ICP-MS analyses. ^**^ calculated values: B_2_O_3_ was calculated for B = 3.00 apfu (details see text); Li_2_O was calculated for a completely filled *Y* site (3.00 apfu); H_2_O was calculated for (OH) + F = 4.00. B_2_O_3_ values as measured by LA-ICP-MS for the following samples are within an error of ≤8% consistent with the calculated values (ICP-MS B_2_O_3_ (wt%): BRA20: 11.05, BRA21: 10.69, BRA22: 11.04, BRA23: 11.20, BRA26: 11.53, BRA27: 10.58, MOZ19: 11.40, MOZ20: 10.46, MOZ22: 10.86, MOZ23: 10.99). Total Fe and Mn calculated as FeO and MnO (see text)

#### Laser ablation ICP-MS analyses

In all tourmaline crystals, B_2_O_3_, the REE and some additional trace elements (Mg, Cr, Ni, Cu, Zn, Pb, Bi) were analysed by laser ablation-inductively coupled plasma-mass spectrometry (LA-ICP-MS) at Erlangen University. The measurements were undertaken on a 266 nm Nd:YAG Laser of New Wave Research (Merchantek) Products, connected to an Agilent 7500i ICP-MS quadrupole instrument at 1250 W plasma power. Ar was used as carrier gas (1.28 L/min) as well as plasma gas (14.9 L/min) and auxiliary gas (0.9 L/min). Data acquisition was performed in Time Resolved Mode with measurements on the maximum peak and 25 ms integration time for all chosen isotopes but 10 ms for B and Si; 15 s for measuring the background and also 15 s for acquisition time. 3 single spots with a crater size of 40 μm, a repetition rate of 10 Hz and a laser energy at 0,51-0,74 mJ (energy density 43-52 J/cm^2^) were ablated on each tourmaline. Data analysis was performed via GLITTER software (Version 3.0, on-line Interactive Data Reduction for the LA-ICP-MS, Macquarie Research Ltd., 2000), using Si as internal standard (values known from electron microprobe).

Tourmalines (Dravite, Schorl, Elbaite; Dyar et al*.* 2001) supplied from Harvard University were measured as monitors to check for accuracy; the relative standard deviation for B is ≤8 %. External calibration was performed via NIST SRM 610 500 ppm glass supplied from the National Institute of Standards and Technology with the values from Pearce et al. (1997), the reproducibility for NIST SRM 612, 50 ppm glass, measured as unknown sample, 5 to 11 % relative. The raw values for B_2_O_3_ were corrected against the Elbaite monitor crystal containing 10.14 wt% B_2_O_3_, an average of 10.10 wt% determined by PIGE, 10.11 wt% by SIMS and 10.20 wt% by PGNAA (Dyar et al. 2001). Results of the LA-ICP-MS analyses are the mean values of 3 ablated distant spots.

The average major and minor element contents (in wt%) and formula occupancies of the tourmaline crystals analysed by EMPA or LA-ICP-MS are presented in Table 1 and the average trace element contents (in ppm) determined by LA-ICP-MS are given in Table 2.

**Table 2:** Trace element contents of Cu- and Mn-bearing fluor-elbaite from Brazil (BRA) and Mozambique (MOZ) (ppm) from LA-ICP-MS

	BRA20	BRA21	BRA22	BRA23	BRA24	BRA25	BRA26	BRA27	BRA28	MOZ19	MOZ20	MOZ21	MOZ22	MOZ23	MOZ24
Mg	0.540	0.862	1.056	4.61	6.95	1.89	441	1199	<0.59	<0.49	<0.34	<0.37	3.36	0.706	1.038
Cu	2632	6460	7066	10141	14180	13532	7559	12550	2063	2362	2767	1506	1189	1365	964
Zn	<1.7	1.59	2.89	92.6	197	14.5	5818	5045	<2.1	65.7	3.81	7.63	1.84	7.18	<2.5
La	0.051	0.036	0.037	1.17	0.512	1.55	0.690	0.202	<0.02	0.026	<0.02	<0.02	<0.02	<0.02	<0.04
Ce	0.067	0.0445	0.041	1.19	0.539	1.53	0.828	0.110	<0.02	<0.02	0.044	<0.02	0.035	<0.02	<0.04
Pr	<0.02	<0.01	<0.01	0.066	0.042	0.116	0.055	<0.02	<0.02	<0.02	<0.01	<0.02	<0.02	<0.02	<0.04
Nd	<0.09	<0.09	<0.09	<0.11	<0.08	0.234	0.141	<0.05	<0.13	<0.12	<0.09	<0.09	<0.11	<0.09	<0.18
Pb	95.4	3.24	3.49	17.8	56.4	48.5	77.5	14.7	20.7	1329	78.4	9.63	25.6	7.82	8.73
Bi	1363	28.5	28.2	112	817	396	2897	115	162	339	792	41.7	73.7	24.6	141

Elements generally below the detection limit (ppm) are: Ni <0.5; Cr <1.3; Sm, Gd < 0.20; Eu < 0.04; Tb, Ho, Tm, Lu < 0.02; Dy, Er, Yb < 0.12

### Crystal structure

The tourmaline fragments were studied on a Bruker AXS Kappa APEX II CCD diffractometer equipped with a monocapillary optics collimator and graphite-monochromatized Mo*K*α radiation. Single-crystal X-ray diffraction data were collected at room temperature (up to 80° 2θ), integrated and corrected for Lorentz and polarization factors and absorption correction by evaluation of partial multiscans. The structure was refined with SHELXL-97 (Sheldrick 1997) using scattering factors for neutral atoms and a tourmaline starting model from Ertl et al. (2008). The H atom bonded to the O3 atom was located from a difference-Fourier map and subsequently refined. Refinement was performed with anisotropic displacement parameters for all non-hydrogen atoms. Table 3 provides crystal data and details of the structure refinement. Site occupancies were refined according to well-known characteristics of the tourmaline structure (Na was refined at the *X* site, Al and Li were refined at the *Y* site; for other details see Table 4). The refinements converged at *R*1(*F*) values of ~1.4-2.1 % (Table 3). The atomic parameters and equivalent isotropic displacement parameters are listed in Table 4. In Table 5 we present selected interatomic distances.

**Table 3:** Sample parameters and refinement results for Cu- and Mn-bearing fluor-elbaite from Brazil (BRA) ad Mozambique (MOZ)

	BRA21	BRA24	BRA26	BRA27	MOZ19	MOZ20	MOZ21	MOZ24
*a*, *c* (Å)	15.820(2), 7.093(1)	15.828(2), 7.098(1)	15.866(2), 7.112(1)	15.869(2), 7.115(1)	15.832(2), 7.102(1)	15.862(2), 7.114(1)	15.864(2), 7.113(1)	15.818(2), 7.095(1)
*h, k, l* ranges	-25/28, -28/28, -12/12	-26/28, -28/28, -12/12	-25/25, -28/28, -12/12	-24/28, -28/22, -12/12	-28/26, -28/24, -11/12	-27/24, -28/27, -12/12	-24/27, -28/27, -12/12	-27/26, -28/28, -12/12
Total reflections measured	18,638	19,307	18,909	20,762	19,462	19,891	18,862	19,467
Unique reflections	2245	2273	2116	2282	2209	2279	2175	2209
2θ_max_ (°)	80°	79.96°	79.97°	79.99°	79.92°	79.96°	79.87°	79.90°
*R*1*(*F*), *wR*2^†^(*F* ^2^)	1.36 %, 3.44 %	1.94 %, 4.43 %	1.86 %, 4.75 %	2.12 %, 4.93 %	1.62 %, 3.99 %	1.90 %, 4.58 %	1.72 %, 4.19 %	1.54 %, 3.70 %
*R*_int_^‡^ (%)	2.44 %	2.40 %	2.44 %	2.95 %	2.40 %	2.07 %	2.04 %	2.97 %
Flack *x* parameter	0.04(5)	0.12(7)	0.01(7)	-0.05(8)	0.09(6)	-0.01(7)	0.05(6)	0.03(5)
'Observed' refls. [*F*_o_ > 4σ (*F*_o_)]	2226	2199	2082	2229	2184	2252	2147	2172
Extinct. coefficient	0.0035(2)	0.0000(2)	0.0160(4)	0.0035(2)	0.0044(2)	0.0012(2)	0.0015(2)	0.0029(2)
No. of refined parameters	95	95	95	95	95	95	95	95
Goodness-of-Fit^§^	1.108	1.100	1.090	1.123	1.125	1.085	1.107	1.078
Δσ_min_, Δσ_max_ (e/Å^3^)	-0.67, 0.77	-1.10, 1.36	-1.08, 1.54	-1.05, 1.89	-0.80, 1.15	-0.88, 1.75	-0.73, 1.07	-0.48, 0.68

X-ray radiation: MoKα (λ = 0.71073 Å); *Z*: 3; space group: *R*3*m* (no. 160); multi-scan absorption correction; refinement on *F* ^2^. Frame width, scan time, detector distance: 2°, 60 s, 35 mm. Scan mode: sets of ϖ and θ scans

* *R*1 = Σ| |*F*_o_| – |*F*_c_| | / Σ|*F*_o_|

^†^ *wR*2 = {Σ[*w*(*F*_o_^2^ – *F*_c_^2^)^2^] / Σ[*w*(*F*_o_^2^)^2^]}^1/2^

*w* = 1 / [σ^2^(*F*_o_^2^) + (*aP*)^2^ + *bP*], *P* = [2*F*_c_^2^ + Max(*F*_o_^2^,0)] / 3

^‡^ *R*_int_ = Σ|*F*_o_^2^ – *F*_o_^2^(mean)| / Σ[*F*_o_^2^]

^§^ GooF = *S* = {Σ[*w*(*F*_o_^2^ – *F*_c_^2^)^2^] / (*n*–*p*)}^1/2^

**Table 4:** Positional parameters and *U*_*equivalent*_ for atoms of Cu- and Mn-bearing fluor-elbaite from Brazil (BRA) ad Mozambique (MOZ)

Site	BRA21	BRA24	BRA26	BRA27	MOZ19	MOZ20	MOZ21	MOZ24
*X*								
*x*	0	0	0	0	0	0	0	0
*y*	0	0	0	0	0	0	0	0
*z*	0.2530(2)	0.2549(2)	0.2539(2)	0.2537(3)	0.2550(2)	0.2547(2)	0.2522(2)	0.2531(2)
*occ.*	Na_0.64(1)_	Na_0.82(1)_	Na_0.84(1)_	Na_0.86(1)_	Na_0.84(1)_	Na_0.88(1)_	Na_0.72(1)_	Na_0.62(1)_
*U*_*eq*_	0.0225(4)	0.0165(4)	0.0218(5)	0.0215(5)	0.0139(3)	0.0183(4)	0.0237(5)	0.0216(5)
*Y*								
*x*	0.12256(3)	0.12372(4)	0.12367(4)	0.12372(4)	0.12346(4)	0.12372(4)	0.12282(3)	0.12208(3)
*y*	½ *x*	½ *x*	½ *x*	½ *x*	½ *x*	½ *x*	½ *x*	½ *x*
*z*	-0.34637(5)	-0.34786(7)	-0.35108(6)	-0.35148(7)	-0.34748(7)	-0.35169(7)	-0.34936(6)	-0.34481(6)
*occ.*	Al_0.715(3)_Li_0.285_	Al_0.697(4)_Li_0.303_	Al_0.890(4)_Li_0.110_	Al_0.973(4)_Li_0.027_	Al_0.592(3)_Li_0.408_	Al_0.780(4)_Li_0.220_	Al_0.782(3)_Li_0.218_	Al_0.622(3)_Li_0.378_
*U*_*eq*_	0.0069(1)	0.0070(1)	0.0071(1)	0.0074(1)	0.0068(1)	0.0074(1)	0.0078(1)	0.0067(1)
*Z*								
*x*	0.29674(1)	0.29697(2)	0.29730(2)	0.29744(2)	0.29683(2)	0.29723(2)	0.29702(2)	0.29661(2)
*y*	0.25990(1)	0.26003(2)	0.26049(2)	0.26055(2)	0.25997(2)	0.26042(2)	0.26027(2)	0.25989(2)
*z*	-0.37157(3)	-0.37098(4)	-0.37013(4)	-0.36974(4)	-0.37099(3)	-0.36998(4)	-0.37066(3)	-0.37173(3)
*occ.*	Al_1.00_	Al_1.00_	Al_1.00_	Al_1.00_	Al_1.00_	Al_1.00_	Al_1.00_	Al_1.00_
*U*_*eq*_	0.00542(4)	0.00567(5)	0.00539(5)	0.00546(6)	0.00543(4)	0.00573(5)	0.00580(5)	0.00541(4)
*T*								
*x*	0.19184(1)	0.19191(2)	0.19200(2)	0.19195(2)	0.19193(1)	0.19196(2)	0.19190(1)	0.19183(1)
*y*	0.18980(1)	0.18986(2)	0.18994(2)	0.18991(2)	0.18989(1)	0.18994(2)	0.18988(2)	0.18978(1)
z	0.01955(2)	0.01903(3)	0.01943(3)	0.01938(4)	0.01882(3)	0.01896(3)	0.01937(3)	0.01943(3)
*occ.*	Si_1.00_	Si_1.00_	Si_1.00_	Si_1.00_	Si_1.00_	Si_1.00_	Si_1.00_	Si_1.00_
*U*_*eq*_	0.00448(3)	0.00480(4)	0.00451(5)	0.00446(5)	0.00451(4)	0.00463(4)	0.00481(4)	0.00457(4)
B								
*x*	0.10909(3)	0.10901(5)	0.10930(5)	0.10932(5)	0.10901(4)	0.10928(5)	0.10929(4)	0.10909(4)
*y*	2*x*	2*x*	2*x*	2*x*	2*x*	2*x*	2*x*	2*x*
*z*	0.4737(1)	0.4739(2)	0.4738(2)	0.4741(2)	0.4735(2)	0.4739(2)	0.4736(2)	0.4733(1)
*occ.*	B_1.00_	B_1.00_	B_1.00_	B_1.00_	B_1.00_	B_1.00_	B_1.00_	B_1.00_
*U*_*eq*_	0.0058(1)	0.0059(2)	0.0056(2)	0.0059(2)	0.0057(1)	0.0061(2)	0.0061(2)	0.0058(1)
O1								
*x*	0	0	0	0	0	0	0	0
*y*	0	0	0	0	0	0	0	0
*z*	-0.2020(2)	-0.1979(3)	-0.1983(3)	-0.1965(4)	-0.1992(3)	-0.1972(4)	-0.2007(3)	-0.2025(2)
*occ.*	O_0.53(4)_F_0.47_	F_0.86(6)_O_0.14_	F_0.96(7)_O_0.04_	F_0.94(8)_O_0.06_	F_0.84(5)_O_0.16_	F_0.91(7)_O_0.09_	F_0.64(5)_O_0.36_	O_0.59(4)_F_0.41_
*U*_*eq*_	0.0317(5)	0.028(1)	0.050(1)	0.059(2)	0.0417(8)	0.055(1)	0.061(1)	0.0282(5)
O2								
*x*	0.06032(3)	0.06014(4)	0.06048(4)	0.06055(4)	0.06016(3)	0.06045(4)	0.06055(3)	0.06028(3)
*y*	2*x*	2*x*	2*x*	2*x*	2*x*	2*x*	2*x*	2*x*
*z*	0.5095(1)	0.5072(2)	0.5057(2)	0.5054(2)	0.5067(1)	0.5043(2)	0.5068(1)	0.5094(1)
*occ.*	O_1.00_	O_1.00_	O_1.00_	O_1.00_	O_1.00_	O_1.00_	O_1.00_	O_1.00_
*U*_*eq*_	0.0131(1)	0.0147(2)	0.0164(2)	0.0168(2)	0.0148(2)	0.0174(2)	0.0160(2)	0.0129(1)
O3								
*x*	0.26394(7)	0.26631(9)	0.26700(9)	0.2679(1)	0.26596(8)	0.26769(9)	0.26566(8)	0.26355(7)
*y*	1/2*x*	1/2*x*	1/2*x*	1/2*x*	1/2*x*	1/2*x*	1/2*x*	1/2*x*
*z*	-0.47322(9)	-0.4728(1)	-0.4724(1)	-0.4719(2)	-0.4730(1)	-0.4721(1)	-0.4726(1)	-0.4736(1)
*occ.*	O_1.00_	O_1.00_	O_1.00_	O_1.00_	O_1.00_	O_1.00_	O_1.00_	O_1.00_
*U*_*eq*_	0.0125(1)	0.0129(2)	0.0118(2)	0.0117(2)	0.0123(1)	0.0120(2)	0.0127(2)	0.0121(1)
H3								
*x*	0.260(2)	0.261(2)	0.265(2)	0.264(2)	0.261(2)	0.263(2)	0.262(2)	0.259(2)
*y*	1/2*x*	1/2*x*	1/2*x*	1/2*x*	1/2*x*	1/2*x*	1/2*x*	1/2*x*
*z*	0.414(3)	0.416(5)	0.407(4)	0.415(5)	0.407(5)	0.416(4)	0.410(4)	0.418(4)
*U*_*iso*_	0.037(6)	0.039(8)	0.022(7)	0.025(8)	0.048(9)	0.028(8)	0.033(7)	0.045(7)
O4								
*x*	0.09395(3)	0.09343(4)	0.09348(4)	0.09331(4)	0.09338(3)	0.09324(4)	0.09368(3)	0.09392(3)
*y*	2*x*	2*x*	2*x*	2*x*	2*x*	2*x*	2*x*	2*x*
*z*	0.09272(9)	0.0919(1)	0.0915(1)	0.0913(2)	0.0921(1)	0.0914(1)	0.0918(1)	0.0928(1)
*occ.*	O_1.00_	O_1.00_	O_1.00_	O_1.00_	O_1.00_	O_1.00_	O_1.00_	O_1.00_
*U*_*eq*_	0.0084(1)	0.0086(2)	0.0085(2)	0.0083(2)	0.0083(1)	0.0084(1)	0.0088(1)	0.0085(1)
O5								
*x*	0.18742(5)	0.18676(8)	0.18689(8)	0.18686(8)	0.18667(6)	0.18664(8)	0.18725(7)	0.18757(6)
*y*	1/2*x*	1/2*x*	1/2*x*	1/2*x*	1/2*x*	1/2*x*	1/2*x*	1/2*x*
*z*	0.11508(9)	0.1148(1)	0.1143(1)	0.1139(2)	0.1144(1)	0.1136(1)	0.1143(1)	0.1154(1)
*occ.*	O_1.00_	O_1.00_	O_1.00_	O_1.00_	O_1.00_	O_1.00_	O_1.00_	O_1.00_
*U*_*eq*_	0.0087(1)	0.0089(1)	0.0087(2)	0.0084(2)	0.0085(1)	0.0085(1)	0.0090(1)	0.0086(1)
O6								
*x*	0.19509(3)	0.19568(4)	0.19626(5)	0.19659(5)	0.19554(4)	0.19634(5)	0.19583(4)	0.19492(4)
*y*	0.18454(3)	0.18515(5)	0.18582(5)	0.18618(5)	0.18511(4)	0.18613(5)	0.18544(4)	0.18433(4)
*z*	-0.20609(6)	-0.20613(9)	-0.20569(9)	-0.2053(1)	-0.20613(8)	-0.20555(9)	-0.20579(8)	-0.20623(7)
*occ.*	O_1.00_	O_1.00_	O_1.00_	O_1.00_	O_1.00_	O_1.00_	O_1.00_	O_1.00_
*U*_*eq*_	0.00716(7)	0.0074(1)	0.0073(1)	0.0073(1)	0.00725(8)	0.0075(1)	0.00781(9)	0.00720(8)
O7								
*x*	0.28652(3)	0.28635(4)	0.28614(5)	0.28599(5)	0.28626(4)	0.28593(5)	0.28618(4)	0.28654(4)
*y*	0.28615(3)	0.28597(4)	0.28605(5)	0.28596(5)	0.28593(4)	0.28585(4)	0.28608(4)	0.28619(3)
*z*	0.09740(5)	0.09785(8)	0.09874(8)	0.09910(9)	0.09796(7)	0.09888(8)	0.09822(7)	0.09718(6)
*occ.*	O_1.00_	O_1.00_	O_1.00_	O_1.00_	O_1.00_	O_1.00_	O_1.00_	O_1.00_
*U*_*eq*_	0.00625(6)	0.00662(9)	0.0064(1)	0.0064(1)	0.00621(8)	0.00644(9)	0.00668(9)	0.00634(7)
O8								
*x*	0.20944(3)	0.20934(5)	0.20953(5)	0.20961(5)	0.20945(4)	0.20967(5)	0.20958(4)	0.20945(4)
*y*	0.27003(3)	0.26982(5)	0.27011(5)	0.27018(6)	0.26993(4)	0.27027(5)	0.27024(4)	0.27005(4)
*z*	0.45806(6)	0.45859(8)	0.45939(9)	0.4601(1)	0.45861(7)	0.45975(9)	0.45889(8)	0.45784(7)
*occ.*	O_1.00_	O_1.00_	O_1.00_	O_1.00_	O_1.00_	O_1.00_	O_1.00_	O_1.00_
*U*_*eq*_	0.00728(7)	0.0076(1)	0.0075(1)	0.0076(1)	0.00740(8)	0.0078(1)	0.00789(9)	0.00716(8)

Definition for *U*_*eq*_ see Fischer and Tillmanns (1988)

**Table 5:** Selected interatomic distances (Å) in Cu- and Mn-bearing fluor-elbaite from Brazil (BRA) and Mozambique (MOZ)

BRA21	BRA24	BRA26	BRA27	MOZ19	MOZ20	MOZ21	MOZ24	
*X*- O2(x3)	2.458(1)	2.435(2)	2.444(2)	2.445(2)	2.432(1)	2.431(2)	2.459(2)	2.457(2)
O5(x3)	2.748(1)	2.746(1)	2.753(1)	2.754(1)	2.747(1)	2.754(1)	2.753(1)	2.749(1)
O4(x3)	2.814(1)	2.811(1)	2.817(1)	2.813(1)	2.810(1)	2.813(1)	2.816(1)	2.813(1)
Mean	2.673(1)	2.664(1)	2.671(1)	2.671(1)	2.663(1)	2.666(1)	2.676(1)	2.673(1)
*Y*- O2(x2)	1.9549(6)	1.9638(8)	1.9653(8)	1.9668(9)	1.9665(7)	1.9684(8)	1.9632(7)	1.9577(6)
O1(F1)	1.9669(6)	2.0022(13)	2.0170(14)	2.0267(18)	1.9934(12)	2.0240(16)	1.9912(12)	1.9537(10)
O6(x2)	1.9677(6)	1.9735(8)	1.9999(8)	2.0074(9)	1.9739(7)	2.0056(8)	1.9942(7)	1.9623(6)
O3	2.1358(10)	2.1464(13)	2.1501(13)	2.1591(14)	2.1477(11)	2.1552(13)	2.1494(12)	2.1425(11)
Mean	1.991(1)	2.004(1)	2.016(1)	2.022(1)	2.004(1)	2.021(1)	2.009(1)	1.989(1)
*Z*- O6	1.8622(5)	1.8558(7)	1.8553(7)	1.8533(8)	1.8565(6)	1.8526(7)	1.8590(6)	1.8634(6)
O7	1.8811(5)	1.8822(7)	1.8803(7)	1.8803(8)	1.8835(6)	1.8824(7)	1.8828(6)	1.8822(5)
O8	1.8852(5)	1.8857(7)	1.8849(7)	1.8843(8)	1.8866(6)	1.8843(7)	1.8861(6)	1.8864(5)
O8’	1.9012(5)	1.9043(7)	1.9091(7)	1.9092(8)	1.9031(6)	1.9072(7)	1.9074(6)	1.9001(6)
O7’	1.9447(5)	1.9467(7)	1.9480(7)	1.9499(8)	1.9491(6)	1.9513(7)	1.9495(6)	1.9440(5)
O3	1.9583(4)	1.9535(6)	1.9624(6)	1.9601(6)	1.9554(5)	1.9589(6)	1.9634(5)	1.9603(5)
Mean	1.9055(5)	1.9047(7)	1.9067(7)	1.9062(8)	1.9057(6)	1.9061(7)	1.9080(6)	1.9061(5)
*T*- O6	1.6048(5)	1.6024(7)	1.6052(7)	1.6025(8)	1.6018(6)	1.6011(7)	1.6057(6)	1.6055(6)
O7	1.6090(5)	1.6086(7)	1.6114(7)	1.6117(7)	1.6086(5)	1.6099(7)	1.6118(6)	1.6092(5)
O4	1.6193(3)	1.6204(4)	1.6232(4)	1.6226(5)	1.6217(4)	1.6230(4)	1.6224(4)	1.6196(3)
O5	1.6336(3)	1.6360(5)	1.6376(5)	1.6368(5)	1.6361(4)	1.6370(5)	1.6371(4)	1.6344(4)
Mean	1.6167(4)	1.6169(6)	1.6194(6)	1.6184(6)	1.6171(5)	1.6178(6)	1.6193(5)	1.6172(5)
B- O2	1.360(1)	1.361(2)	1.361(2)	1.359(2)	1.360(1)	1.359(1)	1.360(2)	1.361(1)
O8(x2)	1.380(1)	1.380(1)	1.381(1)	1.382(1)	1.381(1)	1.383(1)	1.382(1)	1.380(1)
Mean	1.373(1)	1.374(1)	1.374(1)	1.374(1)	1.374(1)	1.375(1)	1.375(1)	1.374(1)

## Results

The investigated tourmalines from Brazil and Mozambique are all enriched in Al, contain relatively high amounts of Li and have a pronounced content of MnO (up to ~3.5 wt%), CuO (up to ~1.8 wt%) and F (up to ~1.8 wt%) (Table 1). Hence, they can be classified as Mn- and Cu-bearing fluor-elbaite (Ertl et al. 2010a; Bosi et al. 2011; Henry et al. 2011). The lattice parameters of our samples are typical for the elbaite subgroup (*a* = 15.82-15.87 Å, *c* = 7.09-7.12 Å; Table 3). Because a Mn^2+^-Ti^4+^ intervalence interaction has been observed in Cu-bearing tourmalines from Paraiba, it can be assumed that Mn^2+^ is usually dominant (pers. comm. George Rossman, 2012). Although the pink component in some Mn-bearing tourmalines indicates the presence of some Mn^3+^, we consider the amount of Mn^3+^ only relatively low (see also Ertl et al. 2003). Because we have no spectroscopic data of our samples, we calculated all Mn as Mn^2+^ (Table 1).

**Fig. 3:**
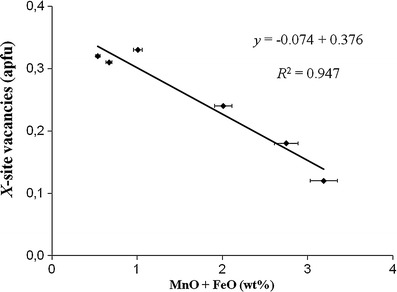
Correlation between *X*-site vacancies and (MnO + FeO) for Cu- and Mn-bearing tourmalines from Brazil with Ca contents ≤0.06 apfu. *Horizontal error bars* show the analytical precision

**Fig. 4:**
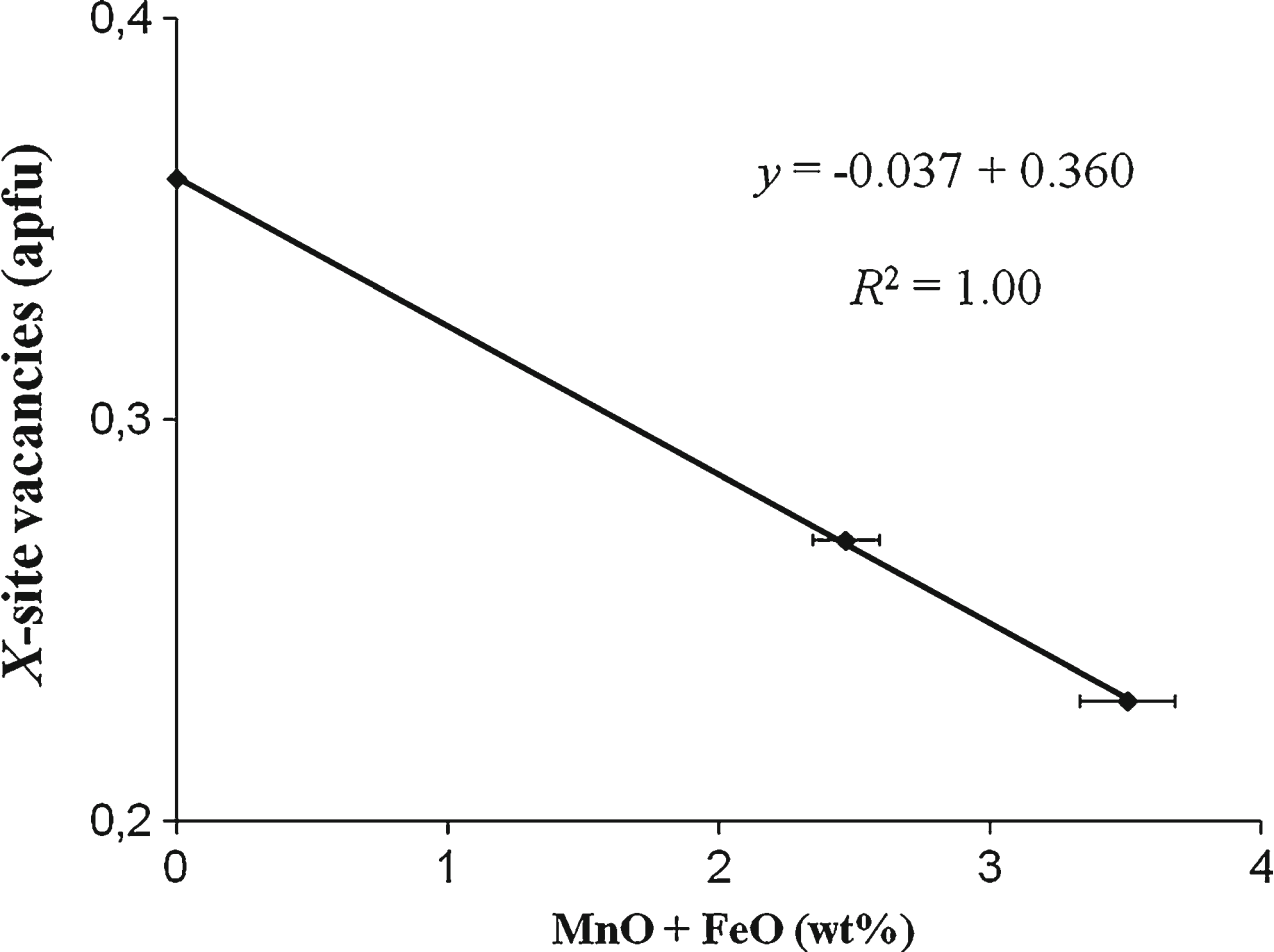
Correlation between *X*-site vacancies and (MnO + FeO) for Cu- and Mn-bearing tourmalines from Mozambique with Ca contents ≤0.02 apfu (For Ca contents ≤0.04 apfu *R* ^2^ = 0.84; 4 samples). *Horizontal error bars* show the analytical precision

**Fig. 5:**
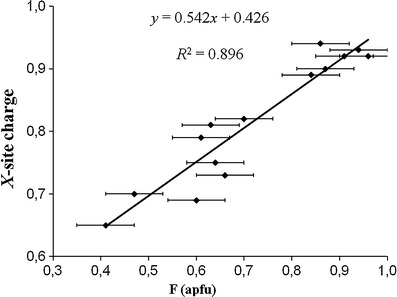
Correlation between *X*-site charges and F content for Cu- and Mn-bearing tourmalines from Brazil and Mozambique. F content from EMPA (Table 1), except for samples BRA21, BRA24, BRA26, BRA27, MOZ19, MOZ20, MOZ21 and MOZ24 (SREF; Table 4). *Horizontal error bars* show the analytical precision, respectively the average standard deviation (±1σ)

**Fig. 6:**
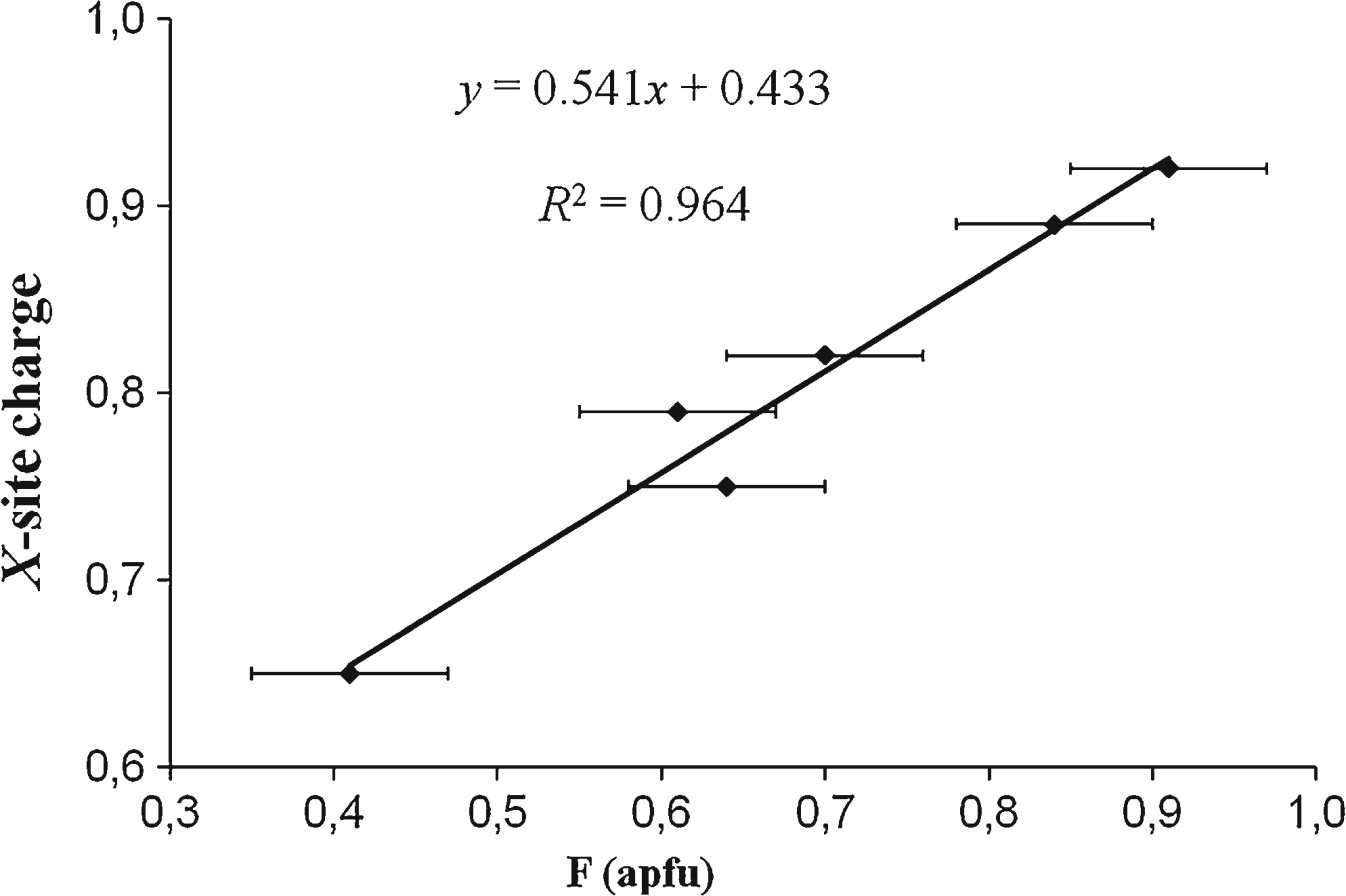
Correlation between *X*-site charges and F content for Cu- and Mn-bearing tourmalines from Mozambique. F content from refinement (Table 4), except for samples MOZ22 and MOZ23 (EMPA; Table 1). *Horizontal error bars* show the average standard deviation (±1σ), respectively the analytical precision

The *X* site in all samples is mainly occupied by Na (0.63–0.82 apfu; Table 1) and is partly vacant (0.12-0.36 apfu vacancies). Significant amounts of Ca (0.01-0.14 apfu) and minor amounts (≤0.01 apfu) of K, Bi and Pb also occupy the *X* site (Table 1, 2). The <*X*-O> distance varies from 2.663(1) to 2.676(1) Å (Table 5). There is a pronounced negative correlation (*R* ^2^ = 0.947; Fig. 3) between *X*-site vacancies and (MnO + FeO) for the tourmalines from Brazil with Ca contents ≤0.06 apfu. A similar correlation (*R* ^2^ = 1.00; Fig. 4) has been observed for the samples from Mozambique, which have Ca contents ≤0.02 apfu. An excellent positive correlation (*R* ^2^ = 0.896; Fig. 5) exists between *X*-site charges and F content for all investigated tourmalines. By plotting only the tourmalines from Mozambique the correlation is significantly improved (*R* ^2^ = 0.964; Fig. 6).

**Fig. 7:**
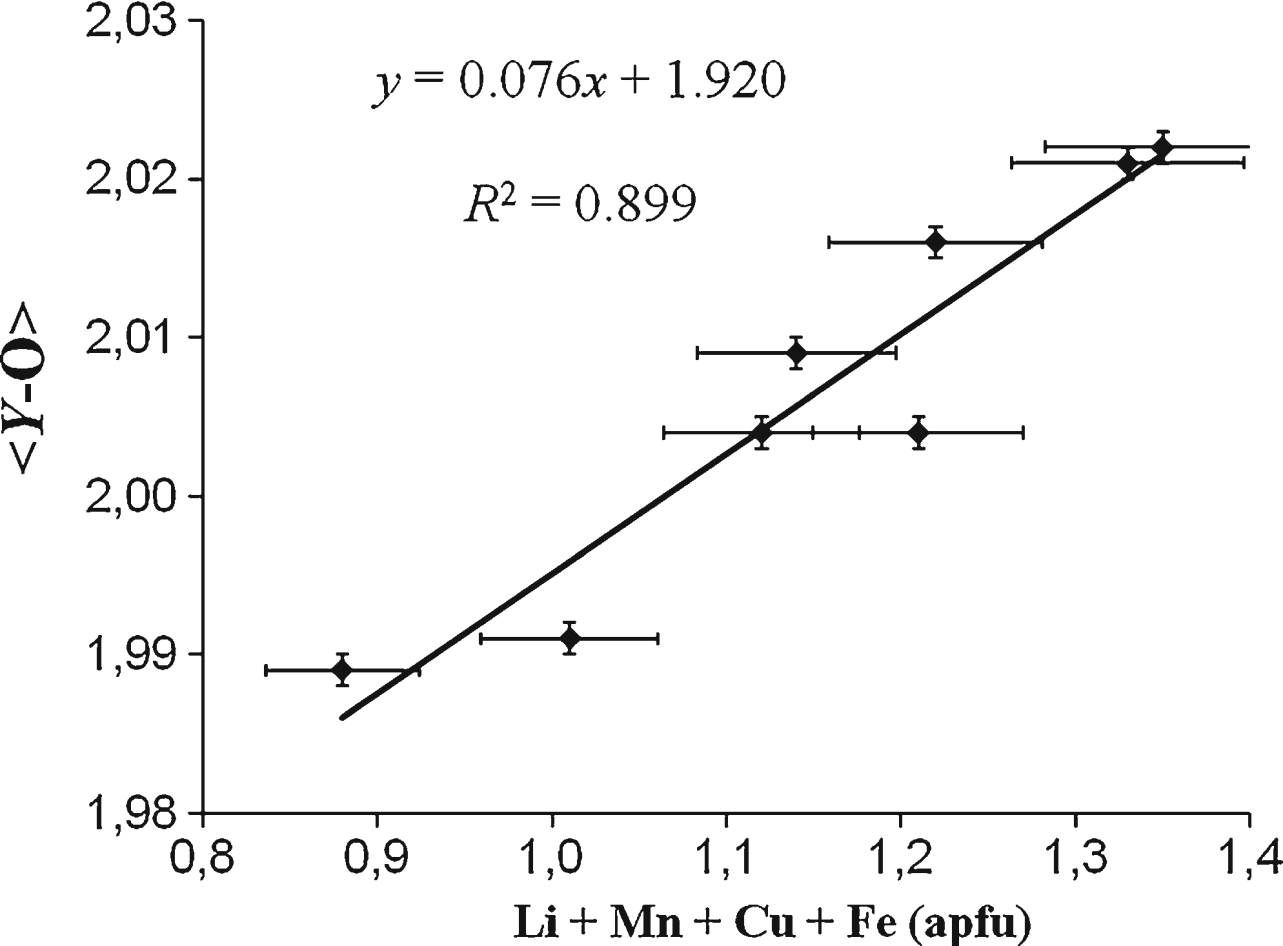
Correlation between the <*Y*-O> distances and the (Li + Mn + Cu + Fe) content (apfu) for Cu- and Mn-bearing tourmalines from Brazil and Mozambique. *Vertical error bars* show the average standard deviation (±1σ), horizontal error bars show the analytical precision

**Fig. 8:**
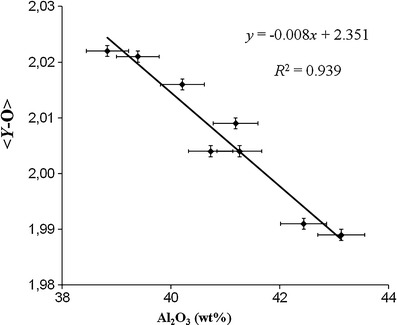
Correlation between the <*Y*-O> distances and the Al_2_O_3_ content for Cu- and Mn-bearing tourmalines from Brazil and Mozambique. Vertical error bars show the average standard deviation (±1σ), *horizontal error bars* show the analytical precision

The *Y* site is mainly occupied by Al (~1.5-2.1 apfu) and Li (calculated Li content: ~0.7-1.0 apfu; Table 1). Significant amounts of Mn^2+^ (≤0.45 apfu) and Cu^2+^ (≤0.2 apfu) also occupy this site. A few samples contain minor amounts of Zn (≤0.09 apfu), Mg (≤0.05 apfu) and Fe^2+^ (≤0.03 apfu). Because Li was calculated, small amounts of vacancies at the *Y* site cannot be excluded. The <*Y*-O> distance varies from 1.989(1) to 2.022(1) Å (Table 5). There is a pronounced positive correlation (*R* ^2^ = 0.899; Fig. 7) between the <*Y*-O> distances and the (Li + Mn^2+^ + Cu + Fe^2+^) contents (apfu) for all structurally investigated samples from Brazil and Mozambique. The influence of the Cu content in this correlation is less significant than that of the other cations (Li, Mn^2+^, Fe^2+^), because the effective ionic radius of Al is less different to Cu than to the other cations (the same correlation as in Fig. 7, but without Cu would result in *R* ^2^ = 0.871). A negative correlation, which is even better (*R* ^2^ = 0.939; Fig. 8), exists between the <*Y*-O> distances and the Al_2_O_3_ content for the tourmalines from both localities.

In all samples the *Z* site is only occupied by Al. Releasing the *Z*-site occupancy during refinement showed the result that this site is occupied by Al_1.00_ within a standard deviation of ±1σ. Hence, in our investigated samples there is no clear evidence for measurable amounts of heavier elements (Mn, Zn, Cu) than Al and the occupancy of the *Z* site was fixed at Al_1.00_ (full occupancy) during the final refinement. The <*Z*-O> distance in all samples is ~1.906 Å within a standard deviation of ±3σ (Table 5). Nevertheless, there exists a positive correlation (*R* ^2^ = 0.71) between the <*T*-O> and the <Z-O> distances for the tourmalines from both localities.

**Fig. 9:**
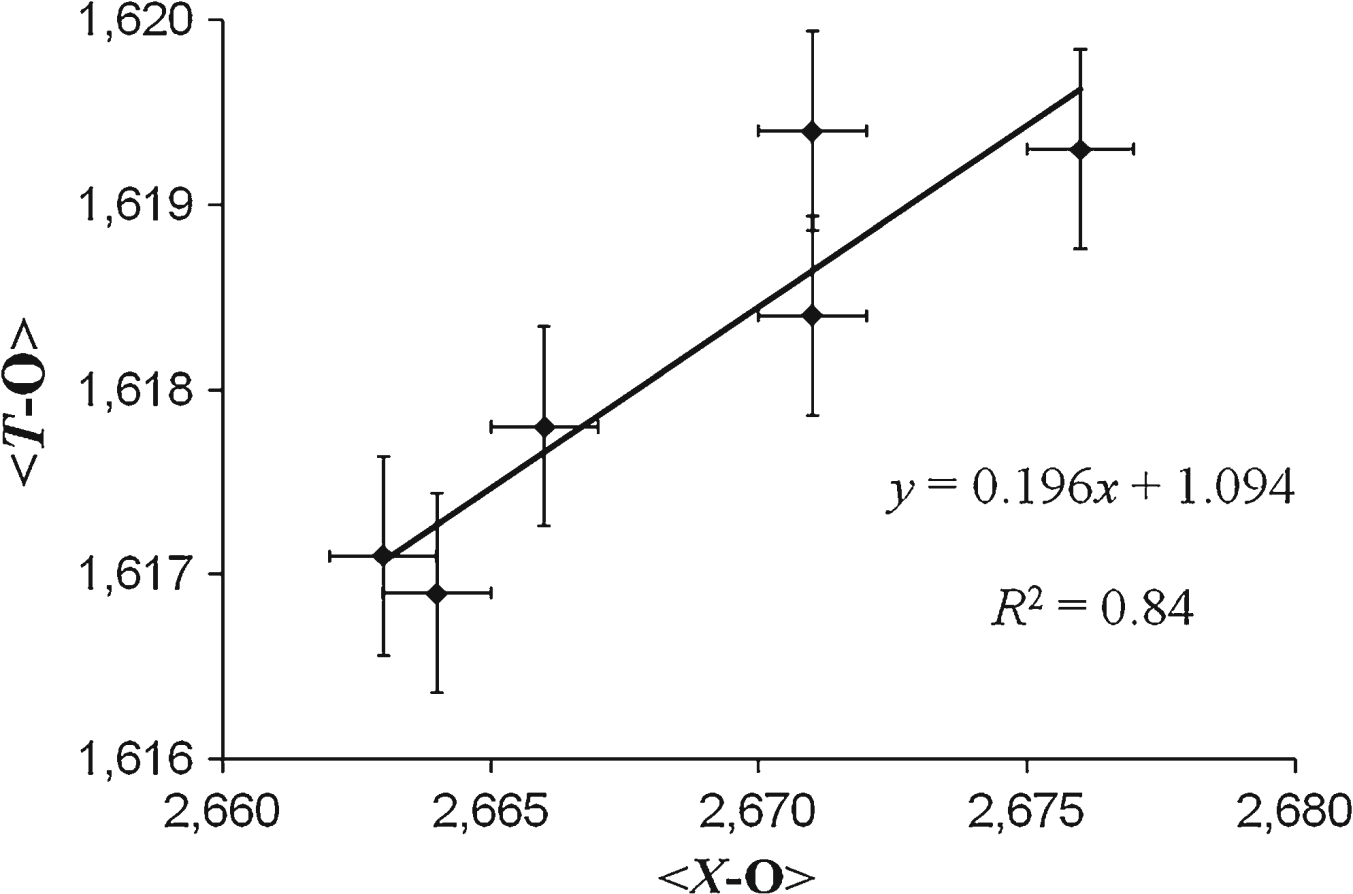
Correlation between the <*T*-O> and the <*X*-O> distances for all Cu- and Mn-bearing tourmalines from Brazil and Mozambique with ≥0.65 apfu F. *Error bars* show the average standard deviation (±1σ)

In all samples the *T* site is essentially occupied by Si (Table 1). Releasing the *T*-site occupancy during refinement did not show a clear evidence for significant amounts of ^[4]^B (>0.10 apfu) in the investigated samples. Hence it was fixed at Si_1.00_ (full occupancy) during the final refinement. The final *T*-site occupancy, which was calculated by using the chemical data, gives up to ~0.1 apfu ^[4]^Al (Table 1). Because of the uncertainty of the chemical analysis of SiO_2_ there is no final prove for the occurrence of ^[4]^Al in our samples. However, some evidence for minor amounts of ^[4]^Al shows only the crystal structure of sample BRA26, because it has the largest <*T*-O> distance of all investigated samples (1.619(1) Å; Table 5). An excellent positive correlation (*R* ^2^ = 0.84; Fig. 9) is observed between the <*T*-O> and the <*X*-O> distances for all structurally characterized samples (with >0.60 apfu F) from Brazil and Mozambique.

The B site in all samples is completely occupied by B (Table 1) and for all structurally investigated samples the <B-O> distance is 1.374(1) Å (Table 5).

**Fig. 10:**
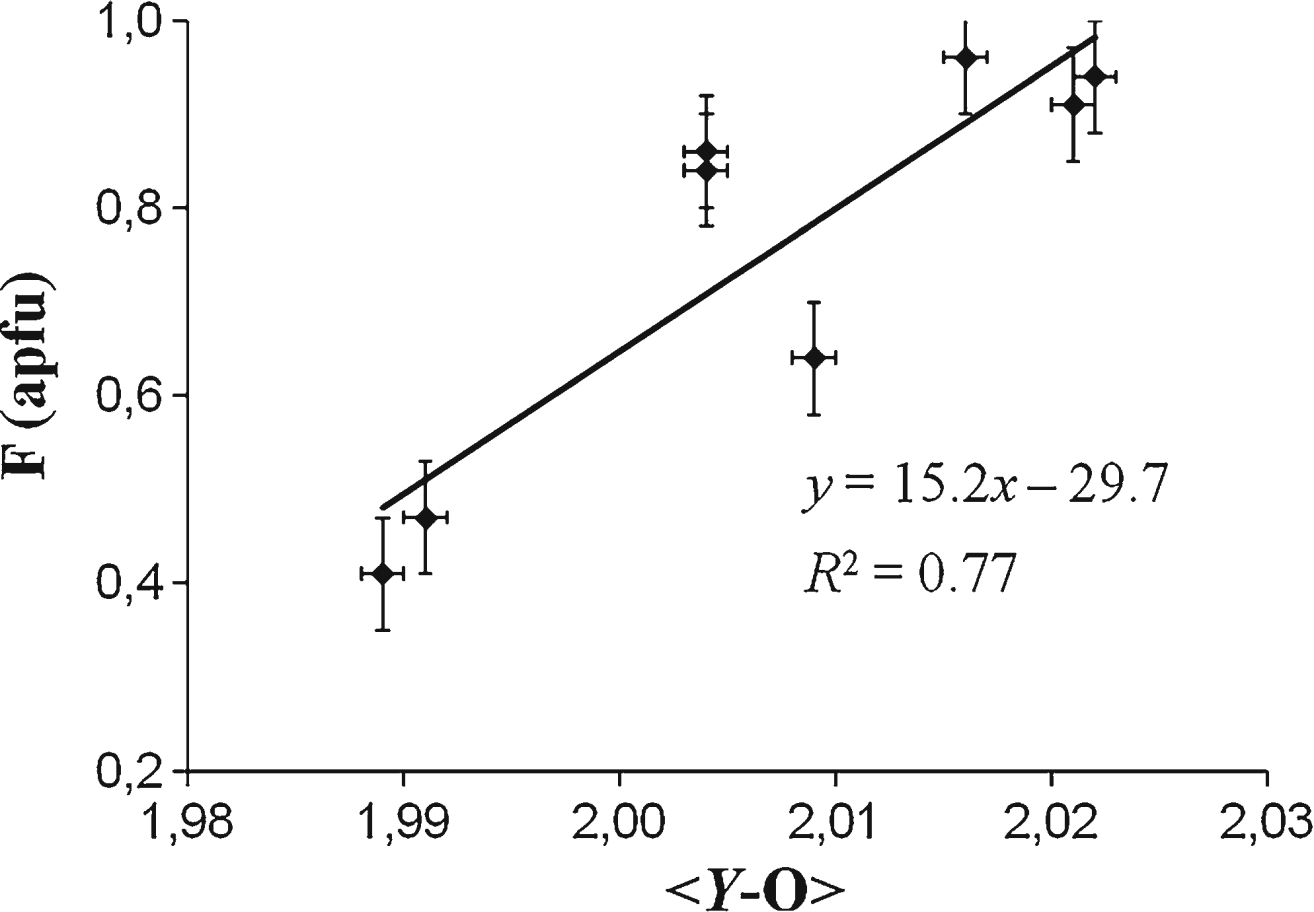
Correlation between the <*Y*-O> and the F content from refinement for Cu- and Mn-bearing tourmalines from Brazil and Mozambique. *Error bars* show the average standard deviation (±1σ)

The *V* site in all samples is occupied by (OH)_3_ and the *W* site shows a relatively high F content in all samples (~0.5-1.0 apfu F; Tables 1, 4). The OH was calculated as OH = 4 – F, because this calculation can be used for elbaitic samples with FeO + MnO < 8 wt% (Ertl et al. 2010a). A positive correlation (*R* ^2^ = 0.77; Fig. 10) is evident between the F content (from refinement) and the <*Y*-O> distances for all structurally characterized samples from Brazil and Mozambique.

## Discussion

Henry and Dutrow (1996) pointed out that, in metamorphic tourmaline, the *X*-site vacancies decrease from ~0.60 to ~0.05 apfu as temperature increases from 200 to >750 °C. They also found that tourmalines that did not exceed metamorphic temperatures of 450 °C contain little or no ^[4]^Al whereas, in high-*T* rocks (with *T* > 750 °C), ^[4]^Al progressively increases up to ~0.25 apfu. Because of a pronounced positive correlation (*R* ^2^ = 0.99) between (Fe^2+^ + Mn^2+^) and ^[4]^Al in tourmalines of the elbaite-schorl series from the Himalaya Mine, Mesa Grande, California, USA (Ertl et al. 2010a), an increase of (Fe^2+^ + Mn^2+^) with increasing temperature can be considered. Our elbaitic tourmalines show an excellent negative correlation between (MnO + FeO) and the *X*-site vacancies (Figs. 3, 4). Because our samples contain only very small amounts of FeO (≤0.2 apfu; Table 1), we conclude that the Mn content in these elbaitic tourmalines depends on the availability of Mn, on the formation temperature, as well as on stereochemical constraints. A few samples with higher Ca contents were excluded from these correlations (Figs. 3, 4), because we consider these samples possibly to be influenced by the host rocks during a late-stage infusion of host-rock components. Pegmatitic tourmalines, which were enriched in Ca due to an interaction between pegmatites and host rocks, are well known (e.g., Ertl et al. 2006, 2010a, and b). Because of a very weak correlation between MnO and CuO (*R* ^2^ = 0.01) in our samples we believe that the Cu content in tourmaline is essentially dependent on the availability of Cu as well as on stereochemical constraints.

In the context of running investigations, a larger number of 52 differently coloured tourmalines were analyzed. Colours range from violet, pink-violet and reddish-violet to violet-blue,blue, blue-green, pale blue-grey, pale blue-green and yellow-green. No obvious correlation of the colour and the content of Cu and/or Mn is recognized. Instead of that, the Cu-content seems to depend primarily on the provenance, with Cu-contents <4,000 ppm in tourmalines from Mozambique and 7,000 – 14,000 ppm in tourmalines from Brazil. Additional spectroscopy is planned to investigate the question of tourmaline colours more in detail.

A pronounced positive correlation (*R* ^2^ = 1.00) between *X*-site charge and F content in tourmaline, first described by Ertl et al. (2010a), was also recorded in our samples (Figs. 5, 6). However, compared to the equation for the elbaite-schorl tourmalines from the Himalaya Mine (*y* = 0.78*x* + 0.37; Ertl et al. 2010a), the equation for our Cu- and Mn-bearing fluor-elbaites is significantly different (*y* = 0.54*x* + 0.43; *R* ^2^ = 0.90-0.96; Figs. 5, 6). We consider these equations to be dependent on the tourmaline compositions, which evolve in a (relatively) closed pegmatitic system at special temperature conditions during the cooling path.

The <*Y*-O> distances in our tourmalines increase with increasing (Li + Mn^2+^ + Cu + Fe^2+^) content (*R* ^2^ = 0.90; Fig. 7) and decrease with an increasing Al_2_O_3_ content (*R* ^2^ = 0.94; Fig. 8). Hence, just by knowing the Al_2_O_3_ content the <*Y*-O> distance can be predicted without having structural data within an error of ≤0.005 Å (<*Y*-O> = 2.3514 - 0.0084 Al_2_O_3_; <*Y*-O> [Å], Al_2_O_3_ [wt%]). Similar correlations were already described in tourmalines of the elbaite-schorl series for the Cruzeiro Pegmatite, Minas Gerais, Brazil (<*Y*-O> to ^*Y*^Al, *R* ^2^ = 0.96; Bosi et al. 2005b) and for various localities (<*Y*-O> to (^*Y*^Al + 0.4Fe^3+^), *R* ^2^ = 0.98; Ertl et al. 2010a).

A positive correlation has been observed between <*T*-O> and <*X*-O> distances for our samples from Brazil and Mozambique which contain ≥0.65 apfu F (*R* ^2^ = 0.84; Fig. 9). Because the <*T*-O> distances do not vary strongly (Table 5), more high quality structural data are necessary for a final prove of this correlation. However, the *X*O_9_ polyhedron is connected to the *T*O_4_ tetrahedron through two oxygen atoms (O4, O5), which could be a possible explanation for such a correlation. A relationship with the F content is evident through crystal-chemical reasoning. The *W* site, located on the three-fold axis central to the pseudohexagonal ring of tetrahedra, is bonded to three *Y*-site cations. In cases OH occupies the *W* site, the H atom points toward the *X* site. Crystallographic studies as well as extensive analytical data on tourmaline establish that F is found exclusively at the *W* site (as summarized by Henry and Dutrow 1996). The presence or absence of the fluorine immediately adjacent to the polyhedron thus may affect the *X*O_9_ polyhedron. Already Henry (2005) and Henry and Dutrow (2011) showed in an evaluation of a large amount of chemical analyses of different tourmalines that, with more than 0.5 *X*-site vacancies, there is little or no F present in the tourmaline. Further publications have shown that there exists a pronounced negative correlation between the number of vacancies at the *X* site and the F content (e.g., Ertl et al. 2009, 2010a). Henry and Dutrow (1996) suggested, with increasing metamorphic grade, an increasing amount of ^[4]^Al (*via* the Al_2_(R^2+^Si)_–1_ exchange vector) and of F contents, and a decrease of *X*-site vacancies *via* the ^*X*^□Al(NaR^2+^)_–1_ exchange vector (R^2+^ = Fe^2+^, Mn^2+^, Mg). The positive correlation between F contents and <*Y*-O> distances (Fig. 10) is perhaps an indication that tourmalines, which crystallized at a higher temperature (because of the inverse relation between *X*-site vacancies and F content), exhibit a larger <*Y*-O> distance. Hence, such tourmalines would be enriched in cations with a larger effective ionic radius (Mn^2+^, Fe^2+^, Li) and depleted in Al_2_O_3_.

Cu-bearing tourmalines from Brazil exhibit relatively low Pb contents (up to ~95 ppm; Table 2) and sometimes significant amounts of Mg (up to ~1200 ppm; Table 2). Cu-bearing tourmalines from Mozambique contain in some cases relatively high amounts of Pb (up to ~1330 ppm; Table 2) and only relatively low Mg contents (up to ~3 ppm; Table 2).

## Conclusion

Blue, bluish green, yellowish green, green and violet-pink tourmalines from Brazil and Mozambique have been characterized chemically and structurally. All these samples can be classified as Mn^2+^- and Cu-bearing fluor-elbaite. Different correlations by using structural and chemical data have been plotted and discussed. We conclude that the excellent negative correlation, which exists between the <*Y*-O> distances and the Al_2_O_3_ content, can be used to predict the <*Y*-O> bond-length, when no crystal structure analysis was performed. The samples at each locality generally show a strong negative correlation mainly between the *X*-site vacancies and the MnO content. We conclude that the Mn content in these tourmalines depends on the availability of Mn, on the formation temperature, as well as on stereochemical constraints. Because of a very weak correlation between MnO and CuO we argue that the Cu content in tourmaline is essentially dependent on the availability of Cu and on stereochemical constraints. Cu contents are <4,000 ppm in tourmalines from Mozambique and in the range of 7,000–14,000 ppm in tourmalines from Brazil. Within the analytical errors Cu and Mn^2+^ occupy only the [6]-coordinated *Y* site. In all investigated tourmalines the *Z* site is only occupied by Al. The *X* site in all samples is mainly occupied by Na, but significant amounts of Bi (up to ~2,900 ppm) and Pb (up to ~1,330 ppm) have also been observed. Cu-bearing tourmalines from Mozambique, compared with samples from Brazil, can have higher amounts of Pb, while tourmalines from Brazil can contain higher contents of ^*Y*^Mg.
